# Complete genome sequences of *Pseudomonas aeruginosa* clone C strains 8277, PT31M, and SG50M isolated from the urinary tract and anthropogenic water environments

**DOI:** 10.1128/mra.01240-25

**Published:** 2026-03-16

**Authors:** Soojeong Ham, Ute Römling, Changhan Lee

**Affiliations:** 1 Department of Biological Sciences, Ajou University 34919, Suwon, Republic of Korea; 2 Department of Microbiology, Tumor and Cell Biology, Karolinska Institutet, Stockholm, Sweden; Loyola University Chicago, Chicago, Illinois, USA

**Keywords:** *Pseudomonas aeruginosa*, clone C, 8277, SG50M, PT31M

## Abstract

We report the complete genome sequence of three *Pseudomonas aeruginosa* clone C strains—8277 (urinary tract), PT31M (drinking water), and SG50M (swimming pool). These genomes broaden available reference strains beyond cystic fibrosis isolates, enabling studies on the ecological adaptability, pathogenic diversity, and global success of clone C.

## ANNOUNCEMENT

**Fig 1:**
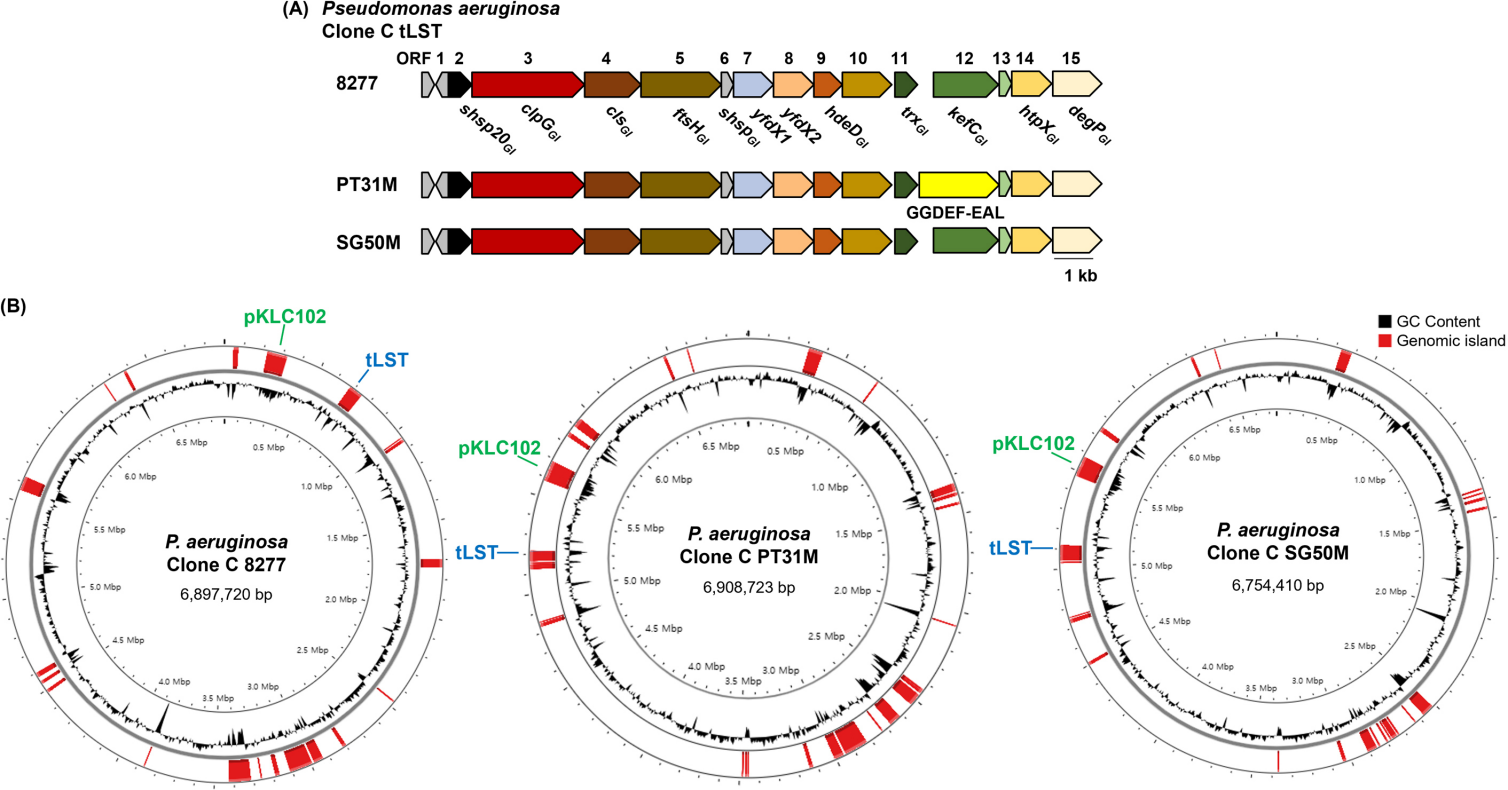
Genomic structure of *Pseudomonas aeruginosa* clone C strains 8277, PT31M, and SG50M with tLST locus. (**A**) tLST maps of the three clone C strains illustrate conservation of gene order across the locus. (**B**) Whole-genome maps display GC content profile (colored in black) and predicted genomic islands (colored in red). Location of tLST and pKLC102 associated with genomic islands is indicated in blue and green, respectively. Genomic maps with GC content profile and genomic island were visualized by Proksee (12).

*Pseudomonas aeruginosa* is an opportunistic pathogen responsible for diverse infections, including chronic lung infections in cystic fibrosis (1). Beyond its clinical significance, the species thrives in the environment, including in diverse natural and anthropogenic aquatic environments, including rivers, drinking water, and swimming pools (2). One of the most widespread lineages, clone C, is found worldwide in aquatic habitats and causing acute and chronic infections (3–5). The success of clone C has been linked to transmissible loci conferring stress tolerance (tLST) harboring molecular chaperones, such as ClpG_GI_ disaggregase and small heat shock proteins (sHsp20_GI_) (4, 6, 7) (Fig. 1A). Genomic plasticity among clone C strains has been previously documented, yet complete genome sequences remain biased toward CF lung isolates, with only a few environmental strains characterized (8–11).

To overcome this bias, we obtained the complete genome sequences for three clone C strains from currently underrepresented niches that had previously been characterized only by pulsed-field gel electrophoresis or partial sequencing. Strain 8277, isolated from the urinary tract in Durham, United Kingdom (4, 13, 14), represents the complete genome from a non-respiratory human infection. Strains SG50M (swimming pool water) and PT31M (drinking water) were collected from anthropogenic aquatic environments in Mülheim an der Ruhr, Germany (4). Prior to this study, partial genome shotgun sequencing data were available only for PT31M in NCBI under accession NZ_NSSQ00000000. These isolates expand the ecological and clinical diversity of clone C genomes, enabling comparative analyses across respiratory vs. urinary tract infection and natural vs anthropogenic water systems.

Genomic DNA was extracted from overnight grown cell cultured in LB in 37°C with shaking and then purified by AccuPrep Genomic DNA Extraction Kit (Bioneer, cat. no. K-3032). Whole-genome sequencing was performed using the PacBio SMRT Sequel and Illumina NovaSeq 6000 platforms; PacBio libraries were prepared from sheared, 7–10 kb size-selected DNA, whereas Illumina libraries were generated by random fragmentation and adapter ligation for paired-end 151 bp (PE151) sequencing. Illumina reads are mapped to the initial PacBio assembly to identify and correct residual sequencing errors. *De novo* assemblies were generated using CANU (v1.7) or SMRT (v8.0) and polished with Pilon (v1.21) (15, 16). Genome annotation was conducted by the PGAP pipeline (v6.10) from NCBI (17).

The complete genomes of strains 8277, PT31M, and SG50M were assembled into single circular chromosomes, as confirmed by terminal sequence overlap, and no plasmids were detected (Table 1, Fig. 1B). Genomes ranged from 6.75 to 6.90 Mb with an average G+C content of 66.7% and contained 6,156–6,318 predicted coding sequences. Each strain carried tLST located in genomic island predicted by IslandViewer four using DIMOB method (4, 6, 7, 18). In addition, the plasmid pKLC102, commonly found in clone C strains (11, 19), was determined to be integrated into the chromosome with mobile element.

**TABLE 1:** Genome sequencing, assembly, and annotation statistics information of *P. aeruginosa* strains PT31M, SG50M, and 8277

Strains	PT31M	SG50M	8277
Chromosome features			
Total sequence length (bp)	6,908,713	6,754,410	6,897,720
Number of sequences	1	1	66.2
N50	6,908,713	6,754,410	6,897,720
GC content (%)	66.1	66.2	66.2
Depth	86.5	88.4	126.1
Genome annotation			
Number of CDS	6,298	6,156	6,318
Number of rRNAs	12	12	12
Number of tRNAs	74	75	75
PacBio sequencing			
No. of subreads	86,070	87,695	127,076
Total no. of subread bases	697,625,891	697,910,834	1,010,030,729
Average read length (bp)	8,105	7,958	7,948
N50	10,675	10,703	10,481
Coverage (×)	101	103	146
Illumina sequencing			
Raw data set			
Total read bases (bp)	3,126,617,778	3,175,056,766	2,914,658,474
Total reads	20,706,078	21,026,866	19,302,374
GC (%)	66.26	67.1	66.83
Filtered data set			
Total read bases (bp)	1,754,529,912	1,803,894,982	1,774,329,278
Total reads	11,623,572	11,951,988	11,754,716
GC (%)	66.22	67.06	67
Mapped reads	11,622,353	11,951,969	11,754,716
Coverage (%)	100	100	100
Depth	242.07	253.18	242.62

These three genomes not only broaden the ecological and clinical range of clone C but also highlight shared and divergent genomic islands beyond tLST. Such differences provide a basis for dissecting niche-specific adaptation and the global success of clone C.

## Data Availability

The whole-genome data have been deposited at GenBank under the accession numbers CM129832 (PT31M), CM129833 (SG50M), and CP170200 (8277). The associated BioProject and BioSample accession numbers are PRJNA1321591 and SAMN51219634 (PT31M), PRJNA1321598 and SAMN51219831 (SG50M), and PRJNA1162667 and SAMN43818467 (8277), respectively. The PacBio and Illumina sequencing raw data for the whole-genome data have been deposited in the NCBI Sequence Read Archive database under the accession numbers SRX30956583 and SRX31989920 (PT31M), SRX30956584 and SRX32015471 (SG50M), and SRX30950279 and SRX32015960 (8277), respectively.
